# Stereotactic radiosurgery for treating meningiomas eligible for complete resection

**DOI:** 10.1186/s13014-021-01748-y

**Published:** 2021-01-28

**Authors:** Maximilian I. Ruge, Juman Tutunji, Daniel Rueß, Eren Celik, Christian Baues, Harald Treuer, Martin Kocher, Stefan Grau

**Affiliations:** 1 Department of Stereotactic and Functional Neurosurgery, Centre for Neurosurgery, Medical Faculty of the University of Cologne, Kerpener Strasse 62, LFI Gebäude Ebene 2, 50937 Cologne, Germany; 2 Department of Radiation Oncology and Cyberknife Centre, Medical Faculty of the University of Cologne, Cologne, Germany; 3 Department of General Neurosurgery, Centre for Neurosurgery, Medical Faculty of the University of Cologne, Cologne, Germany

**Keywords:** Meningioma WHO grade I, Stereotactic radiosurgery (SRS), Local control, Regional control, Adverse events

## Abstract

**Background:**

For meningiomas, complete resection is recommended as first-line treatment while stereotactic radiosurgery (SRS) is established for meningiomas of smaller size considered inoperable. If the patient´s medical condition or preference excludes surgery, SRS remains a treatment option. We evaluated the efficacy and safety of SRS in a cohort comprising these cases.

**Methods:**

In this retrospective single-centre analysis we included patients receiving single fraction SRS either by modified LINAC or robotic guidance by Cyberknife for potentially resectable intracranial meningiomas. Treatment-related adverse events as well as local and regional control rates were determined from follow-up imaging and estimated by the Kaplan–Meier method.

**Results:**

We analyzed 188 patients with 218 meningiomas. The median radiological, and clinical follow-up periods were 51.4 (6.2–289.6) and 55.8 (6.2–300.9) months. The median tumor volume was 4.2 ml (0.1–22), and the mean marginal radiation dose was 13.0 ± 3.1 Gy, with reference to the 80.0 ± 11.2% isodose level. Local recurrence was observed in one case (0.5%) after 239 months. The estimated 2-, 5-, 10- and 15-year regional recurrence rates were 1.5%, 3.0%, 6.6% and 6.6%, respectively. Early adverse events (≤ 6 months after SRS) occurred in 11.2% (CTCEA grade 1–2) and resolved during follow-up in 7.4% of patients, while late adverse events were documented in 14.4% (grade 1–2; one case grade 3). Adverse effects (early and late) were associated with the presence of symptoms or neurological deficits prior to SRS (*p* < 0.03) and correlated with the treatment volume (*p* < 0.02).

**Conclusion:**

In this analysis SRS appears to be an effective treatment for patients with meningiomas eligible for complete resection and provides reliable long-term local tumor control with low rates of mild morbidity.

## Introduction

The incidence of meningioma is increasing due to both an aging population and increased use of MR and CT imaging [1]. Due to their frequent slow growth, meningiomas can develop to a considerable size before elevated intracranial pressure or local irritations cause symptoms such as neurological deficits, seizures, cognitive impairment or psychiatric abnormalities. However, even small tumors in close proximity to cranial nerves may cause early symptoms or deficits [2].

In terms of management, the options include primary observation for incidental, asymptomatic and small tumors, whereas radical resection is recommended for progressive and symptomatic meningiomas.

Tumor relapse strongly depends on WHO grading as well as the extent of surgical resection, which is graded according to the Simpson classification [3].

According to recent EANO guidelines, surgery is the first choice if therapy is indicated, and aims at radically removing the tumor, including (Simpson grade I), or at least coagulation (Simpson grade II) of the involved dura. In contrast, for tumors in complex locations where surgical treatment is unsuitable in the first place, safety and efficacy of stereotactic radiosurgery have been demonstrated by numerous publications [4, 5].

In an aging population with a corresponding impaired general medical condition, the benefits of surgery have to be critically balanced against risks associated with this intervention. Additionally, some patients refuse surgical treatment for personal reasons. For such patients, SRS may therefore provide a suitable treatment option.

We therefore analyzed a unique cohort of patients with surgically accessible meningiomas (generally ≤ 3 cm in diameter) who usually are not presented for radiosurgery in clinical daily practice. We assessed adverse effects and tumor control, and discuss these results in the context of comparable surgical data.

## Methods

### Patient selection

For this single center retrospective analysis, approved by the Ethics Committee of the University of Cologne (approval no. 16-476), all consecutive patients with intra-cranial meningiomas eligible for a presumed Simpson Grade I or II resection with a minimal clinical and radiological follow-up period of six months who underwent SRS between 1994 to 2019 were selected from our radiosurgery database. The suitability for radical resection (Simpson grade I or II [3]) was independently assessed by two experienced neurosurgeons (SG and MIR).

For all patients, SRS treatment was indicated: (1) when pre-therapeutic subsequent MR imaging (minimal interval 12 months) showed typical slow growth dynamics, and imaging patterns suggested a WHO Grade I meningioma, or (2) for recurrences after microsurgical resection of histologically proven meningiomas WHO I. All therapeutic options were discussed within an interdisciplinary neuro-oncological board comprising neurosurgeons, neurosurgeons specialized in radiosurgery, neurologists and radiation oncologists. The patients were accordingly advised by both a neurosurgeon and a neurosurgeon specialized in radiosurgery. The decision for SRS was finally made based on a co-morbidity status resulting in an increased peri-operative risk (assessed by the anaesthesiologist in charge, or the respective physicians of the patient) or the explicit wish of the patients.

### Data acquisition

Data were retrieved from an electronic database and patients´ paper charts. We recorded histopathological data in cases of previous resection, neurological symptoms leading to tumor diagnosis, and symptom development until the last clinical follow up. Adverse events (AE) were rated following the Common Terminology Criteria for Adverse Events (CTCAEv4.03), based on assessing subjective complaints (i.e. headache, dizziness, vertigo, etc.) as well as sound objective deficits during clinical follow-up visits. Adverse events were classified as early (within the first six months after SRS treatment) or late.

For the radiological follow-up, a first MRI was conducted six months after treatment and annually thereafter. Any tumor recurrence was classified as local (= recurrence within the therapeutic SRS isodose) or regional (= recurrence neighboring the treated meningioma outside the therapeutic isodose).

Tumor response was assessed according to the MacDonald criteria [6] as follows: partial remission (PR) in the case of a decrease in tumor size ≥ 50%, and progressive disease (PD) in the case of an increase of ≥ 25%. All other conditions were rated as stable disease (SD).

With regard to treatment parameters, the SRS technique (modified LINAC vs. robotic assisted LINAC SRS by Cyberknife), number of fractions, surface dose and isodose level, as well as target coverage, were documented.

### SRS treatment planning and delivery

The tumor and potentially vulnerable adjacent structures (e.g. brainstem, cerebellum, trigeminal nerve) were contoured on a planning CT (Siemens Somatom Plus or Philips MX8000, since 2012 Toshiba Aquilon) and on a defined set of MRI series comprising contrast enhanced T1, T2 weighted  and FLAIR images (Phillips MR-Scanner, 1.5 or 3 T) registered to the planning CT. Contouring was performed by neurosurgeons experienced in radiosurgery.

For patients treated by the modified LINAC, the patient’s head was immobilized under local anesthesia in a stereotactic frame (Riechert-Mundinger). The SRS planning was carried out using the software STP (STP 3.3 and 3.5, Howmedica Leibinger, Freiburg, Germany). Subsequently, the radiosurgical treatment was performed using a linear accelerator as previously described [7, 8]. In brief, dose application was performed with circular collimators fitted to an adapted linear accelerator (Philips SL 75/20 at 9 MV or Elekta Sli25 at 6 MV). An arching beam technique was used, and for individual treatment planning, this standard technique was individually modified in terms of collimator diameter, table position, number of table angles, ranges of gantry rotation, beam weight, irradiation dose, and number of isocenters. For robotic Cyberknife SRS, the patient was immobilized on the robotic Cyberknife treatment table (Accuray, Sunnyvale, California) by means of a custom-made aquaplast mask. For treatment planning, the software Multiplan v4.5 (since 2016 v4.6) was used as described [9]. If tumor size was > 3 cm or > 14 cc, or the target was located in close proximity to vulnerable structures (chiasma, optic nerve) a hypo-fractionated SRS regime was applied with the Cyberknife with 5 × 5 Gy. All other cases received single fraction SRS by the modified LINAC or the Cyberknife. The final irradiation plan was evaluated in an interdisciplinary consensus meeting between the stereotactic neurosurgeon, a radiation oncologist experienced in SRS, and a medical physicist.

### Statistical analysis

For descriptive statistics, continuous values are given in median and range or mean and standard deviation, ordinal and categorical variables are stated in numbers and percentages. Local and regional recurrence rates and impacting covariates were analyzed by the Kaplan–Meier method and univariate Cox Regression analysis. To determine the influence of ordinal or nominal variates on the occurrence of side effects, Chi square statistics was applied. P-values lower than 0.05 were considered statistically significant. Statistical analysis was performed using SPSS Statistics Version 25 (IBM, Chicago IL).

## Results

### Patient parameters

We included 188 patients with 218 meningiomas treated at the University of Cologne between March 1994 and September 2019. Twenty patients were treated for two meningiomas at different locations within one SRS session, one patient was treated for 5 neighboring convexity meningiomas.

For 69 (36.7%) patients either advanced age and/or comorbidities such as cardiovascular, pulmonary, endocrine, hematopoietic and/or coagulopathy diseases influenced the decision against surgery, while 76 (40.4%) patients chose SRS on purpose as an alternative to (re-)resection. In 43 (22.8%) no information about opting for SRS over resection could not be obtained from our records.

The median age was 60.0 (range 22.6–86.7) years, 148 (78.7%) were female. Detailed data regarding the treated patients  is displayed in Table 1.

**Table 1:** Summary of patient and treatment parameters

No. of patients	188	
No. of meningiomas	218	
Sex (f:m) (No., %)	148 (78.7%): 40 (21.3%)	
Age at day of treatment (median, mean, SD, range in years)	60.0, 60.3, 12.6, 22.7–86.6	
Laterality (No. %)	Left 94 (43.1%); right 85 (39.0%);	
	Medial 39 (17.9%)	
Localization (No. %)	Frontal	53 (24.3%)
	Posterior fossa	47 (21.6%)
	Parietal	19 (8.7%)
	Clivus/sellar/foramen magnum	19 (8.7%)
	Olfactrory grove	18 (8.3%)
	Lateral sphenoid	14 (6.4%)
	Medial sphenoid	13 (6.0%)
	Temporal	13 (6.0%)
	Occipital	12 (5.5%)
	Tentorial	9 (4.1%)
	Other	1 (0.5%)
Adverse Events		
Early adverse events (n patients; %)	Headache	9 (4.7%)
	Dizziness/vertigo	4 (2.1%)
	Seizure(s)	3 (1.6%)
	Cranial nerve deficits	2 (1.1%)
	Facial pain	1 (0.5%)
	Visual field deficit	1 (0.5%)
	Other	1 (0.5%)
	All adverse events rated CTCAE Grade 1 or 2	
Late adverse events (n patients; %)	Headache	8 (4.2%)
	Dizziness/vertigo	8 (4.2%)
	Cranial nerve deficits	7 (3.7%)
	Seizure(s)	2 (1.1%)*
	Visual field deficit	1 (0.5%)
	Other	1 (0.5%)
	All adverse events rated CTCAE grade 1 or 2 except *one case with intractable sensible seizures (CTCAE grade 3)	
Irradiation technique (n; %)	Modified LINAC	91 (41.7%)
	Cyberknife	127 (58.3%)
	Single fraction SRS	206 (94.5%)
	Multi fraction SRS*	12 (5.5%)
Irradiation parameters (median; SD; range)	Target volume (ml)	2.9 ± 3.9; 0.1–22.0
	Prescribed dose(Gy)	13.0 ± 3.1; 10.0–25.0*
	Isodose level (%)	80.0 ± 11.1; 33.0–96.0
	Coverage (%)	99.3 ± 3.1; 69.9–100.0
	*(Multi-fraction SRS 5 × 5 Gy)	

Five patients received multiple SRS treatments for local, regional, or distant recurrences (two SRS treatments for two patients and three treatments for three patients).

### Treatment parameters

Single fraction SRS was applied for 94.5% of the meningiomas, and a hypo-fraction SRS regime with the Cyberknife (5 × 5 Gy) was applied for twelve cases. Between 1995 and 2011, modified LINAC (91 treatments) was used, and since 2012 the Cyberknife (127 treatments). Due to proximity to vulnerable structures, we reduced the dose to 10–12 Gy in 61 (27.9%) cases and applied a hypo-fractionionated SRS regime with the Cyberknife (since 2012) in 12 (5.5%) cases. In fifteen cases (6.8%) a dose of 14 Gy was administered and all other 130 (59.6%) cases received 13 Gy. (Table 1).

### Initial presentation

Ninety nine (45.4%) of the 218 meningiomas were detected as incidental findings or related to unspecific symptoms, but had displayed a clear growth dynamic of the suspected meningioma during their follow-up, while 64 (29.4%) had local or regional recurrence after previous resection. The median interval between resection and SRS was 57.1 months (range 5.5–283.9). No patient had received previous external beam radiation therapy.

Self-reported symptoms or sound objective deficits leading to the diagnosis were caused by 55 (25.2%) tumors and comprised cranial nerve impairment in seven (3.2%), dizziness and vertigo in 14 (6.4%), headache in 19 (8.7%), seizures in 3 (1.4%), visual deficits in 1 (0.5%) and other symptoms in 4 (1.8) cases. For seven (3.2%) patients, data regarding symptoms or deficits were missing. All of these symptoms were transient except the three cases suffering from seizures, two cases with cranial nerve deficits, and two cases reporting dizziness. The latter remained unchanged throughout follow-up after SRS, and were therefore not rated as early or late adverse events. In contrast, we did count the three patients suffering from seizures as adverse events, due to an increase in seizure activity in all three at early follow-up (n = 3), but only persistent in two cases at late follow-up.

### Clinical status upon follow-up

The median clinical follow-up period was 55.8 (range 6.2–300.9) months.

In 21 (11.2%) patients early adverse events occurred that were rated mild to moderate (CTCAE grade 1–2), and which had resolved completely at last follow-up in 14 (7.4%) patients.

Seventeen (9.0%) patients developed late adverse events CTCAE Grade 1–2, except one case suffering from intractable sensible seizures (CTCAE Grade 3) after initial resection and SRS treatment for recurrence of a convexity meningioma in the post central sulcus area (Table 1).

Focusing on cranial nerve deficits, after SRS treatment two patients (1.1%) developed early cranial nerve deficits and 7 (3.2) displayed late deficits noted at the last follow-up. One patient’s early cranial nerve deficit resolved; in one patient it persisted until last follow-up.

Temporary steroid treatment due to symptomatic peri-tumoral edema was administered in 7.4% of cases. According to in-house standards dexamethasone was applied with an initial dosage of 4 mg for one week with tapering off according to symptom development. None of the patients received steroids for more than 21 days.

## Radiological follow-up

**Fig. 1:**
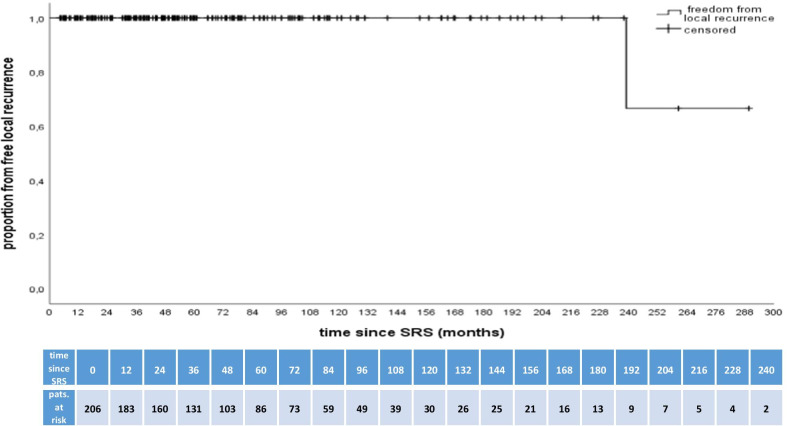
Plot of patients free from local tumor recurrence against time. The Kaplan–Meier estimates freedom from regional recurrent meningiomas. The table below the graph displays the numbers of patients at risk during follow-up after SRS in 12-month intervals

The median radiological follow-up period was 51.4 (range 6.2–289.6) months. During this period, 213 (97.7%) meningiomas were stable, while four (1.8%) showed a partial remission. Local recurrence was observed in one patient after 238.9 months, which was treated by a second, hypo-fractionated SRS with Cyberknife (5 × 5 Gy, 80 isodose level) and remained stable up to 73 months after re-treatment. (Fig. 1).

**Fig. 2:**
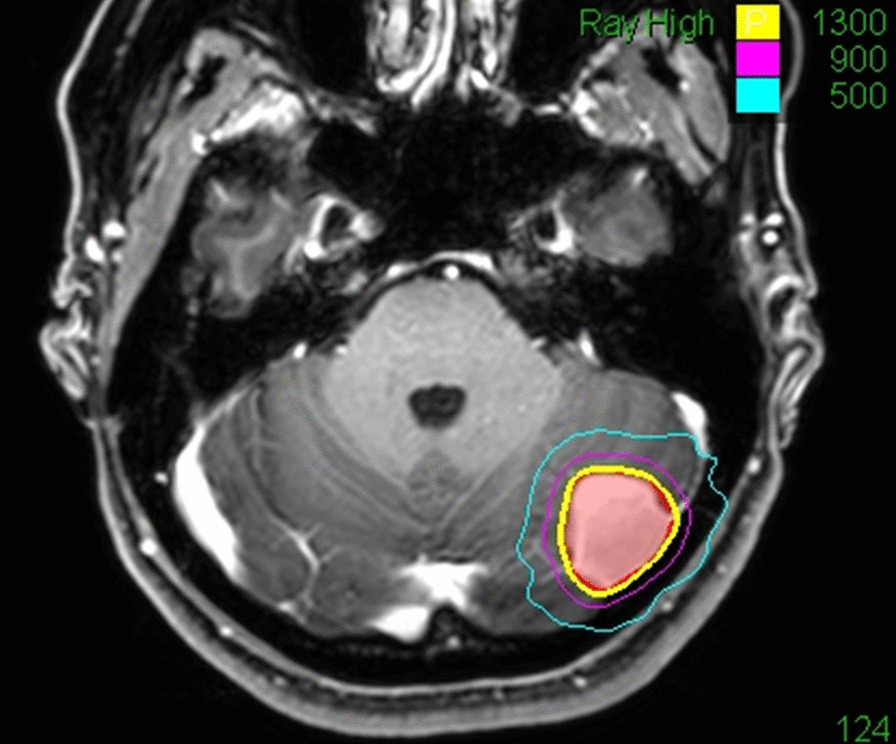
Illustrative case: a 39-year-old female patient underwent MRI for intermittent tinnitus in January 2013 with diagnosis of an incidental convexity meningioma in the posterior fossa. Initially she refused any treatment. A follow-up MRI after 12 months demonstrated tumor progression with 4 mm growth in diameter. She refused microsurgical resection due to religious reasons (Jehovah's Witness) and underwent Cyberknife radiosurgery in April 2014 (single fraction SRS, 13 Gy, 70% isodose level). Throughout her further course until last follow-up in April 2020 she had no subjective complains nor neurological deficits. The annual follow-up MRIs showed stable disease with no reaction of the surrounding tissue. The red line represents the outlined tumour surface, the yellow line the applied 13 Gy surface dose, and the violet and blue lines the 9 Gy and 5 Gy isodose

Regional recurrences occurred in 8 treated patients after 10.6, 10.7, 17.2, 39.2, 40.2, 79.1, 104.6, and 237.9 months. The resulting estimated regional recurrence rates after 2, 5, 10, and 15 years were 1.5%, 3.0%, 6.6% and 6.6%, respectively. All of them received a second SRS treatment. See Fig. 2 as an illustrative case.

### Prognostic factors

Treatment failure was not influenced by the radiosurgical device (p = 0.20) or, in cases of modified LINAC, the collimator used (p = 0.34). Also, neither the target volume (p = 0.45), isodose distribution (p = 0.185), tumor coverage (p = 0.66), nor number of treated lesions (p = 0.59) showed a significant impact.

The appearance of AE was significantly associated with the presence of symptoms or deficits prior to SRS (p < 0.014). Furthermore, AE correlated with treatment volume (p < 0.001), whereas age (p = 0.168), tumor location (p = 0.155), applied marginal dose (0.065) or isodose level (p = 0.973) had no significant influence.

## Discussion

This study reports on treatment outcomes after SRS for meningioma patients, for whom, according to current EANO guidelines, radiosurgery is not the first choice treatment modality, but who rather should undergo surgery [4]. However, this level B recommendation does not originate from the evidence class I data. The composition of the cohort reported here resulted from the presence of co-morbidities and personal preferences in individual patients, which led to the application of radiosurgery instead of surgery.

Here we raise the issue of whether SRS for surgically accessible tumors can match surgical resection in terms of local control and complication rates. Numerous publications, involving large cohorts, document the efficacy of meningioma resection, showing that local control rates for WHO I meningiomas depend mainly on the extent of resection (i.e. Simpson grade [3]). Although most surgical studies include tumors of larger volumes, the reported control rates after Simpson I and II can be used for comparison with the present study. In this context, our local control rates are far above the range of surgical series even after Simpson I and II resections of WHO I tumors [10–13]. For instance, Sughrue et al. [14] reported a 5-year recurrence rate after resection of WHO I meningioma (n = 373) for patients receiving a Simpson Grade I, II, III, or IV resection of 95, 85, 88, and 81%, respectively. In our series the local and regional control rates were at least at such levels, however, since some patients were lost to follow-up, a higher incidence of later tumor relapse is certainly possible. Then again, our long follow-up may substantiated this high range of tumor control, which is in accordance with previous studies reporting 10-year control rates ranging to over 90% [2, 15].

The issue of a mandatory follow-up after meningioma is still unresolved, resulting in highly heterogeneous follow-up schemes of differing quality (ranging from only MRI to physical examination). This is prevalent in both surgical and radio-surgical reports, and therefore leaves guideline recommendations at level IV [4].

Since surgery aims to completely remove tumor tissue and the dura involved, a surgical risk for damage to the CNS exists, but this is also true for radio-surgical procedures. The rates of adverse events in this study are within the range of previous studies and showed a self-limiting course in the vast majority of cases [5, 15–18]. Furthermore, some complaints were not characteristic for tumor location (e.g. headache, dizziness and vertigo) and could not be attributed exclusively to SRS but rather also to co-founding factors e.g. increasing age. In contrast, reported surgical complication rates for asymptomatic meningiomas are clearly higher (ranging from 4 to 13% [19, 20]. However, data analyzing surgical management specifically for small tumors are not available to date. In particular, a large-scale data analysis reported a 30-day-readmission rate for patients after meningioma resection of 10.9% [21]. Furthermore, a recent register-based study from Sweden analyzed sick-leave periods after resection of meningiomas, revealing an unexpectedly high rate of 42.7% of patients not returning to full time occupation two years after surgery [22]. In this context, our complication rate reported here cannot be compared to surgical series in a sound manner, but may well indicate a relatively low incidence compared to lager surgical cohorts. Putting the presented data into perspective with other large SRS series, we are within the range of reported complication rates among larger series of skull base meningiomas that report an incidence of cranial nerve deficits of up to 9.2% [2, 15].

Comparing the impact of treatment on the patients´ life reveals obvious differences between surgery and SRS. While the first includes hospitalization, post-surgical sick leave from work and (depending on the country) restrictions on driving a vehicle, a SRS treatment can be conducted in an outpatient setting without comparable consequences after treatment. Again, no sound comparison between surgical series and this present study is possible, since issues such as quality of life and sick-leave numbers have not been evaluated here, nor do they exist for a corresponding surgical cohort.

With respect to the side effects of treatment using either method, the question of whether any therapy should be indicated at all for incidental meningiomas is highly relevant. In fact, a recent prospective observational study [23] showed a rather high percentage of growing tumors among incidental findings which were, however, of low clinical relevance. A large retrospective study reported a stable tumor size over 5 years in more than 60% of the patients, with only as few as 6% of the patients with growing meningiomas developing any symptoms at all. In this context, the indication for treatment in our series may be questioned, along with most other treatment reports on asymptomatic meningioma.

Nowadays, an increasingly used argument for surgery is to obtain histopathological and molecular diagnoses. This is of particular importance in the light of recent studies that in addition to the WHO grading show a fundamental impact of molecular features on the patient’s further course [24–26]. Such molecular diagnostic data, which are increasingly used in neuro-oncological decision-making, cannot be provided by SRS since it is usually applied without previous tissue analysis. In this context, any assumption of a specific WHO grade of the treated tumors is also speculative. However, the large size of this present cohort and the overall treatment results render a relevant number of higher WHO grades or a significant impact of molecular factors on the course after SRS less probable, but does make long-term radiological follow-up for all patients mandatory; this may be different for surgical patients with a molecular diagnosis.

This study carries all the drawbacks of a retrospective study. However, this cohort was treated and followed-up in a standardized manner in an experienced center with a high caseload. Furthermore, the represented cohort does not reflect the majority of patients presented to a neurosurgical facility, but comprises co-morbid and dismissive patients. The comparability with surgical and other SRS series is limited due to the narrow definition of the presenting tumors with regard to size and speed of growth, but the overall cohort size may give rise to a sound interpretation of the outcome data.

## Conclusion

In our study SRS for small, surgically eligible meningiomas appeared highly effective in achieving local control and did not lead to major complications. According to these results, if treatment is indicated at all, such patients should also be counselled about SRS as a treatment alternative to microsurgery.

## Data Availability

All data generated or analysed during this study are included in this published article. The individual datasets of each patient generated during and/or analysed during the current study are not publicly available because individual privacy could be compromised but data are available from the corresponding author upon reasonable request.
